# Psychometric Properties of the Korean Version of the PsyMate Scale Using a Smartphone App: Ecological Momentary Assessment Study

**DOI:** 10.2196/17926

**Published:** 2020-07-21

**Authors:** Yong Sook Yang, Gi Wook Ryu, Philippe A E G Delespaul, Mona Choi

**Affiliations:** 1 Mo-Im Kim Nursing Research Institute Yonsei University College of Nursing Seoul Republic of Korea; 2 Department of Psychiatry and Neuropsychology, Faculty of Health Medicine and Lifesciences Maastricht University Maastricht Netherlands

**Keywords:** psychometrics, reliability, validity, PsyMate scale, ecological momentary assessment

## Abstract

**Background:**

Ecological momentary assessment (EMA) is a method for capturing the changes in the variables in daily life with increased accuracy and decreased recall bias. The PsyMate scale assesses momentary moods in daily life and can be used in various settings.

**Objective:**

The aim of this study was to develop a Korean version of the PsyMate (K-PsyMate) scale and evaluate its psychometric properties by using the EMA method in patients with moyamoya disease (MMD) in South Korea.

**Methods:**

Patients with MMD aged over 18 years were recruited from July 2018 to January 2019 at the inpatient and outpatient departments of a university hospital in South Korea. The K-PsyMate scale comprising 13 items was developed following a translation/back translation approach of the English version and loaded onto a mobile app. Participants were instructed to enter their moods 4 times a day for 7 consecutive days. Content validity index, factor analysis, and Pearson’s correlation were performed for validity analysis. For reliability, intraclass correlation coefficients between the first and last measurements were estimated by mean rating, absolute agreement, and a 2-way mixed-effects model. Usability was analyzed through a descriptive analysis, 2-tailed t test, and analysis of variance, and the results were confirmed by Mann-Whitney U test and the Kruskal-Wallis test, as the dependent variable was not normally distributed.

**Results:**

In total, 1929 assessments from 93 patients were analyzed. The mean age of the participants was 40.59 (SD 10.06) years, and 66 (71%) of the 93 participants were women. Content validity was excellent as content validity index was 0.99, and 2 factors, negative affect and positive affect, were derived by an exploratory factor analysis. The correlations between the subdomains of the K-PsyMate scale and Hospital Anxiety and Depression Scale were significant (*P*<.001). The agreement between the first and last measurements was poor to moderate according to the obtained intraclass correlation coefficient values. Usability was evaluated by 67 (72%) out of the 93 participants. The participants rated the accuracy of assessing their momentary moods on the app at 4.13 (SD 0.97), easiness in understanding questions, operating, and inputting answers at 4.12 (SD 0.88), and interruption by the survey alarms at 2.48 (SD 1.02) out of 5.

**Conclusions:**

The K-PsyMate scale has good validity but poor to moderate agreement, which reflects the characteristics of the EMA data collected in real and natural living environments without control. The findings of our study show that the K-PsyMate scale uploaded in a mobile app can be a valid and reliable tool for evaluating the momentary mood of patients with MMD because using a mobile app is convenient and patients are familiar with their own smartphones, which they use in their daily lives.

## Introduction

Moyamoya disease (MMD), also called as spontaneous occlusion of the circle of Willis, is a chronic progressive cerebrovascular disease with no known cause and is characterized by fine collateral vessel networks that resemble hazy cloud-like puffs of smoke in the brain [1,2]. MMD occurs worldwide with high prevalence in East Asian countries such as Japan and Korea [3]. Unlike MMD in children, MMD in adults is associated with potential rupture or ischemia of the blood vessels in the brain, similar to that reported in other cerebrovascular diseases [4,5], and this can lead to high mortality [6]. The clinical features of patients with MMD often appear as mental deterioration, headache, and speech impairment [6], which are affected by mood changes. Adults with MMD also show high levels of anxiety and depression [7] and their moods change over time during the course of life [8]. Negative mood and mood changes in adults with MMD may affect their motivation for proper health behaviors and eventually affect the health outcomes of patients with similar cerebrovascular diseases [7]. Therefore, monitoring the moods of adults with MMD can be critical for both patients and health care personnel.

Measuring moods may help people better understand their emotional states. The measuring tool to self-assess moods should be understandable and comfortable to use; at the same time, the tool should be able to assess the mood accurately and capture the fluctuations and changes in the mood reliably since mood varies according to context and time [9,10]. To avoid mood changes caused by the tool itself, the participants should be allowed to respond to the questionnaire in a flexible and friendly atmosphere of their choice.

Traditionally, mood has been assessed by retrospective measures, including pen and paper tests [11], but these methods lead to recall bias and risk of reduced accuracy [9,10]. However, momentary assessments such as ecological momentary assessment (EMA) or experience sampling methods allow increased accuracy and reduced bias because these assessments are performed in real-life environments and through repeated assessments at multiple times [9-11]. The EMA method is known for its effectiveness in capturing changes [12] and understanding daily changes in psychological states such as mood and stress [13,14]. It is broadly used to assess the psychological aspects of the participants with [15-17] and without mental health problems [18]. The increased prevalence of smartphones and advanced mobile technology has made people more familiar with the use of smartphones in their daily lives, and EMA methods using smartphones are available [19-22]. The prevalence of smartphone use in Korean adults is about 95% [23], and about 96.4% of the population under the age of 70 years in Korea can access internet services [24]. In a study conducted with healthy participants in Korea, the EMA method was evaluated as comfortable to use and easy to understand when the mood was assessed using a mobile app installed in their own smartphones [22].

Various measurement tools using mobile technology have been developed to measure moods in daily life. The PsyMate scale was developed to assess momentary moods relating to the daily life of the participants [25], and it has been used in various settings and populations [26,27]. In this study, we aimed to develop a Korean version of the PsyMate (K-PsyMate) scale, upload it on a mobile app to measure the momentary moods of Korean adults with MMD, and examine the psychometric properties of this scale.

## Methods

### Participants

Patients with MMD diagnosed via magnetic resonance angiography and aged over 18 years were recruited from July 2018 to January 2019 at the outpatient department or the inpatient wards of a university hospital in Seoul, South Korea. Patients who could not communicate owing to neurological problems were excluded by a physician and the physician’s assistant who were involved in the treatment of patients with MMD. The Korean version of the Mini-Mental State Examination [28] was used to assess the cognitive impairment of the participants after acquiring the permission to use it from the provider. Participants with a score under 24 in the Korean version of the Mini-Mental State Examination were excluded. Participants who used operating systems other than the Android system were also excluded, since the developed mobile app was available only for the Android operating system as described in the previous study [22]. Informed consent was obtained from all the participants when they enrolled in the study. This study was approved by the Institutional Review Board of the Yonsei University Health System (No. 4-2018-0385).

### Measurements

#### Participants’ Characteristics and Baseline Mood

All participants were asked to provide their demographic and disease-specific information at the baseline measurement through a questionnaire that was developed by our research team. Demographics included age, gender, marital status, people living with, job status, monthly household income, education level, and smoking and drinking history. Detailed information on the participants’ demographics has been presented in our previous work [29]. Regarding the baseline mood, the Hospital Anxiety and Depression Scale (HADS) [30] was administered after obtaining permission to use it. The HADS is known as a globally reliable and valid scale for assessing mental health in hospital settings [31]. It consists of 14 items, 7 for anxiety and 7 for depression, and it is measured on a 4-point scale ranging from 0 to 3. A higher value indicates a higher level of anxiety and depression. In this study, we administered the Korean version of HADS [32], for which Cronbach α was .89 and .86 for anxiety and depression subscales, respectively [32]. The HADS was assessed to compare the momentary negative affect and positive affect that were measured by the K-PsyMate scale, as described in a previous study [25].

#### K-PsyMate Scale for Measuring Momentary Mood

The mood scale of the K-PsyMate was used for the EMA section of this study. This scale consists of 13 items, of which 4 items measure positive affect and 9 items assess negative affect. The positive affect comprised feeling “cheerful,” “satisfied,” “relaxed,” and “globally well,” and the negative affect comprised feeling “lonely,” “guilty,” “worried,” “down,” “threatened,” “insecure,” “irritated,” “frightened,” and “suspicious.” These items were rated on a 7-point scale, with “1” indicating “not at all” and “7” indicating “very much.” Momentary mood was measured while considering the environmental context, taking into account what the participants were doing, where they were, and with whom they were when responding to the questionnaire.

#### Usability Measure

The usability of the EMA method on the mobile phone was evaluated by the participants at the end of the survey. A 10-item questionnaire was uploaded in the app: 2 items for accuracy, 3 items for easiness, 2 items for enjoyment, 2 items for intention, and 1 item for interruption. It was adapted from a previous study [33] with the permission from the developer and scored on a 5-point scale (1=strongly disagree to 5=strongly agree). An open-ended question was included for the participants to offer any additional comment or opinion. The Cronbach α of the scale in this study was .74.

### Procedure

#### Translation of the PsyMate

We freely downloaded the English version of the PsyMate scale [34] with permission from the copyright holder. To develop the K-PsyMate scale, we used the translation/back translation approach. We employed a committee approach for translation, in which we consulted 3 nursing professors who were fluent in both English and Korean to translate the English version of PsyMate [25]. Through this method, we identified language and cultural differences in selecting words. The translated PsyMate items were prepared for back translation during which we consulted 3 different nursing professors who were fluent in both English and Korean. The research team compared the back-translated PsyMate with the original English version to confirm that the translated Korean version was accurate. After confirming the translated Korean version through back translation, the research team finalized the K-PsyMate draft. This draft was then evaluated by a linguist of the Korean language prior to determining the final version. Eventually, the K-PsyMate version was finalized by the research team.

#### Data Collection

While obtaining informed consent from each patient, members of the research team obtained baseline data on demographic and disease-specific information by using the structured questionnaire and on mood status by using the HADS. They helped each participant install the mobile app on their mobile phone and register themselves, and they provided time for practice. Participants selected the EMA sampling period to assess momentary moods according to their convenience. They were guided by an alarm to respond 4 times a day for 7 consecutive days (4 times × 7 days = 28 times/person) at 60-minute blocked semirandom intervals. Notifications were sent in the morning between 8 AM and 9 AM, early afternoon between noon and 1 pm, evening between 5 PM and 6 PM, and at night between 9 PM and 10 PM. A reminder for each scheduled measurement was sent to the participants who did not input the response within 45 minutes after the first notification. The contact numbers and the email addresses of the members of the research team were given to the participants to contact the team if they had any questions or if they faced any difficulties during the assessment period. Finally, patients were requested to complete a 10-item survey at the end of the EMA session on the usability of the EMA method using a mobile app, including an open-ended question.

### Statistical Analysis

Data were analyzed using the SPSS v.25.0 (IBM Corp). We used descriptive statistics to analyze the baseline data of the participants’ characteristics, the HADS scores, and the positive affect and negative affect of the K-PsyMate scale measured by the EMA method. We applied a 2-tailed *t* test and analysis of variance to examine the differences between groups according to the general characteristics. Regarding validity tests, we used the content validity index (CVI), which calculated scale-level CVI (S-CVI) and each item-level CVI (I-CVI). To assess CVI, each item was appraised by an expert panel consisting of 6 nursing professors. The CVI for each item was scored using a 4-point scale, where 1=not relevant, 2=somewhat relevant, 3=quite relevant, and 4=highly relevant. S-CVI and I-CVI were evaluated with the cut-off of S-CVI level at 0.90 and I-CVI at 0.78 [35]. We performed exploratory factor analysis (EFA) to examine construct validity using principal component analysis and varimax rotation. We also used Pearson’s correlation (*r*) to assess the association between the HADS and PsyMate in the previous study [25]. We calculated intraclass correlation coefficients (ICCs) for evaluating the test-retest reliability of the measurement tool. Reliability reflects both the degree of correlation and agreement between the measurements, and ICCs are an index that show the degree of correlation and agreement between the measurements [36]. Among the 10 different forms of ICC [37,38], we calculated the ICCs of each item between the first and the last measurement by mean rating, absolute agreement, and a 2-way mixed-effects model, since we tested absolute agreement of the mean of multiple measurements from a nonrandomly selected population [36]. The obtained ICC values of each item were interpreted by its 95% CI ranges [36]. The ICC estimates in the 95% CI were evaluated by considering ICC<0.50, 0.50≤ICC<0.75, 0.75≤ICC<0.90, and ICC≥0.90 as poor, moderate, good, and excellent reliability, respectively [39]. For usability evaluation, we performed descriptive analysis, 2-tailed *t* test, and analysis of variance, and we confirmed the results by Mann-Whitney *U* test and the Kruskal-Wallis test, as the dependent variable was not normally distributed.

## Results

### Participants

We recruited patients with MMD from the inpatient and outpatient departments of a tertiary hospital in South Korea. We approached 119 patients of which 109 (91.6%) agreed to participate, and they were enrolled using the mobile app. Among the 109 patients, 98 (89.9%) participated in the EMA session. The data of 93 participants who provided more than 3 measurements considered as EMA data [40] and who provided 1929 responses (74.1%) out of the 2604 scheduled assessments were analyzed. The mean age of the participants was 40.59 (SD 10.06) years, and 66 of the 93 participants (71%) were women. The HADS anxiety and HADS depression scores were 7.17 (SD 3.38) and 7.14 (SD 3.51), respectively. The overall mean negative affect and positive affect scores were 2.15 (SD 1.12) and 4.70 (SD 1.31) out of 7, respectively.

### Validity of the K-PsyMate Scale

We calculated S-CVI and I-CVI as evaluated by the expert panel. The S-CVI was 0.99 (I-CVI range 0.83-1.00), which represented an excellent level of content validity. We also calculated the S-CVIs of 2 factors of negative affect and positive affect according to the original study [25], and they were 1.00 and 0.96, respectively. The I-CVIs are presented in Table 1.

EFA was performed based on 1929 completed assessments from 93 participants. We confirmed the presence of latent factors in the observed items with excellent Kaiser-Meyer-Olkin of 0.93 and with significant Bartlett’s sphericity measures (*P*<.05). Two factors were derived with the eigenvalue greater than 1 with a cumulative percentage of explained variance of 71.1%. All items were assigned to either the negative affect (Factor 1) or positive affect (Factor 2) factor. These 2 factors were negatively related (*r*=–0.67). The results of the EFA are shown in Table 2.

The subdomains of negative affect and positive affect of the K-PsyMate scale and the HADS anxiety and depression were significantly correlated with coefficients (*r*) ranging between 0.33 and 0.68. The Cronbach α of the HADS anxiety and depression in this study were .84 and .79, respectively. The correlation coefficients between the K-PsyMate and the HADS subscales are shown in Table 3.

**Table 1:** Item-level content validity index of the PsyMate items (n=1929).

Item number	Items	Item-level content validity index
1	Feeling cheerful	1.00
2	Feeling satisfied	1.00
3	Feeling relaxed	1.00
4	Feeling globally well	0.83
5	Feeling lonely	1.00
6	Feeling guilty	1.00
7	Feeling worried	1.00
8	Feeling down	1.00
9	Feeling threatened	1.00
10	Feeling insecure	1.00
11	Feeling irritated	1.00
12	Feeling frightened	1.00
13	Feeling suspicious	1.00

**Table 2:** Factor loadings from EFA^a^ of the K-PsyMate scale (n=1929).^b^

Item number	Items	Communality	Factor 1: Negative affect	Factor 2: Positive affect
2	Feeling satisfied	0.89	–0.23	*0.91*
3	Feeling relaxed	0.87	–0.26	*0.90*
1	Feeling cheerful	0.81	–0.21	*0.87*
4	Feeling globally well	0.80	–0.32	*0.84*
12	Feeling frightened	0.75	*0.83*	–0.23
10	Feeling insecure	0.75	*0.82*	–0.27
13	Feeling suspicious	0.70	*0.82*	–0.19
9	Feeling threatened	0.69	*0.82*	–0.15
6	Feeling guilty	0.63	*0.78*	–0.16
7	Feeling worried	0.70	*0.77*	–0.33
8	Feeling down	0.65	*0.68*	–0.44
11	Feeling irritated	0.62	*0.62*	–0.48
5	Feeling lonely	0.39	*0.52*	–0.35

^a^EFA: exploratory factor analysis.

^b^Values shown in italics show factor loadings >0.50, indicating that the value fits for the given factor.

**Table 3:** Correlations between the K-PsyMate scale and the HADS^a^ (n=93).

HADS subscales				K-PsyMate subscales		
		Negative affect				Positive affect
**HADS anxiety**						
	*r*	0.465^b^				–0.348^b^
	*P* value	<.001				<.001
**HADS depression**						
	*r*	0.446^b^				–0.328^b^
	*P* value	<.001				<.001

^a^HADS: hospital anxiety and depression scale.

^b^The correlation is significant at a significance level of <.05 (2-tailed).

### Reliability

The calculated ICC values between the first and the last measurements ranged from 0.52 to 0.80. Given the range of the 95% CI of each item, all 4 items of the positive affect factor and 6 items of the negative affect factor showed poor level of agreement. Three items of “down,” “insecure,” and “worried” in the negative affect factors were between 0.50 and 0.75 of the 95% CIs, which was considered as moderate agreement. The ICC value and the 95% CI of each item are shown in Table 4.

**Table 4:** ICC^a^ and 95% CI of the K-PsyMate scale (n=93).

Items		First measure, mean (SD)	Last measure, mean (SD)	ICC	95% CI
**Positive affect**					
	Satisfied	4.37 (1.26)	4.68 (1.41)	0.67	0.497-0.779
	Cheerful	4.43 (1.32)	4.76 (1.31)	0.53	0.293-0.689
	Globally well	4.57 (1.40)	4.65 (1.38)	0.53	0.297-0.691
	Relaxed	4.57 (1.42)	4.74 (1.45)	0.52	0.277-0.682
**Negative affect**					
	Down	2.74 (1.61)	2.43 (1.58)	0.80	0.694-0.866
	Insecure	2.88 (1.74)	2.40 (1.63)	0.73	0.599-0.824
	Worried	3.38 (1.78)	2.71 (1.67)	0.67	0.502-0.781
	Frightened	2.55 (1.62)	2.19 (1.55)	0.66	0.490-0.776
	Lonely	3.01 (1.66)	2.38 (1.62)	0.65	0.477-0.770
	Suspicious	2.33 (1.50)	2.01 (1.41)	0.63	0.435-0.752
	Guilty	2.25 (1.46)	2.04 (1.41)	0.61	0.415-0.743
	Threatened	1.99 (1.33)	1.80 (1.18)	0.60	0.393-0.733
	Irritated	2.72 (1.70)	2.26 (1.49)	0.54	0.327-0.704

^a^ICC: intraclass correlation coefficient.

### Usability of the EMA Method by Using a Mobile App

Usability was evaluated by 67 (72%) out of the 93 participants. The mean age was 42.0 (SD 10.43) years (range, 22-67 years), and 50 (74%) of the 67 participants were females. Of the 67 participants, 26 (39%) were in their thirties, whereas only 4 (6%) were in their sixties (Multimedia Appendix 1). Participants rated the accuracy of inputting their momentary mood on the app at 4.13 (SD 0.97) out of 5. Easiness in understanding questions and in operating and in inputting answers was rated at 4.12 (SD 0.88) out of 5. The enjoyment in participating in the EMA app was rated at 3.65 (SD 0.91) out of 5. The participants rated interruption by the survey alarms at 2.48 (SD 1.02) out of 5. The intention to participate or recommend others to participate in another study using EMA apps was reported as 3.25 (SD 0.95) out of 5 (Table 5).

No difference in the usability was found in the subgroup analysis by gender, age, and marital status of the participants (Multimedia Appendix 1). Of the 67 participants, 20 (30%) wrote answers to the open-ended question in the app. Six (9%) out of 67 participants reported that they experienced discomfort owing to the small checkbox and 1 (1%) experienced network disconnection while inputting responses into the app. One participant (1%) expressed that it was a burden to answer 4 times a day.

**Table 5:** Usability of the EMA^a^ method by using a mobile app (n=67).

Category of rating	Mean (SD) of the ratings	Possible range for the ratings
Accuracy in assessing momentary mood	4.13 (0.97)	1-5
Easiness in understanding questions, operating, and inputting answers	4.12 (0.88)	1-5
Enjoyment of participating via the EMA app	3.65 (0.91)	1-5
Interruption by the survey alarms	2.48 (1.02)	1-5
Intention to participate or recommend others to participate in another study using EMA apps	3.25 (0.95)	1-5

^a^EMA: ecological momentary assessment.

## Discussion

### Principal Results

In this study, we evaluated the psychometric properties of the K-PsyMate scale to measure moods by using the EMA method for adults with MMD since their moods are prone to change when they consciously strive to overcome negative feelings and stress, similar to patients with an unruptured cerebral aneurysm [5]. The content validity of the K-PsyMate scale evaluated by S-CVI was excellent with good I-CVI in all items determined by Polit et al [35]. The EFA results agreed with those of a previous evaluation of the PsyMate [25], which also had 2 factors: positive affect with 4 items and negative affect with 9 items. We found that the statistically significant correlations between the K-PsyMate scale and HADS were also in accordance with those reported in the previous study, which had significant correlations ranging from 0.4 to 0.7 when compared with the HADS scores [25].

In this study, the agreement between the measurements evaluated by the ICC values of each item was poor to moderate. This poor to moderate level of agreement suggests that the measurements may have been affected by context variables since participants were requested to measure in an uncontrolled natural real-life environment [9,29]. This finding is in accordance with the results of a study on the Dutch version of the PsyMate, which presented sensitivity to the change of mood over time and applicability for patients in an ambulatory mental health setting in the Netherlands [25]. The poor ICCs may reflect the dynamics of affect fluctuating and changing over time [41] and the variability in the anxious and depressive mood of patients with MMD [7,42].

Considering the characteristics of the EMA data, we tried a 2-level confirmatory factor analysis [43] as an alternative method, wherein latent variables of negative affect and positive affect were assessed at the individual level. However, it did not achieve convergence, and the maximum log pseudo-likelihood stayed the same at –45854.379 from the 20th iteration. Convergence rates for 2-level confirmatory factor analysis models are known to be predicted by all possible interactions between the reliability condition (poor to moderate level by ICCs in this study), number of clusters (93 individual clusters in this study), and cluster size (ranging from 3 to 28 in this study) [44]. Multilevel confirmatory factor analysis cannot be applied to this study in which individuals form groups of interest with small cluster size and with low ICCs between measurements in single-level analysis.

The result of the usability evaluation in this study suggested that the EMA app has a good feasibility with accuracy in assessing mood and without much interruption and it may be applicable, regardless of gender or age, as no statistical difference of usability was found in the subgroup analysis.

### Limitations

This study has the following limitations. First, we conducted the study by using a limited sample size. However, the psychometric properties of the scale could be analyzed by including 1929 measures obtained through the EMA method. Second, we recruited participants who only used the Android operating system, for which the mobile app was developed [22]. Hence, patients who used other operating systems such as the iPhone operating system were not included in this study. Nevertheless, the study is still meaningful in that we contacted about 1.2% of the target population with MMD in South Korea when considering the overall prevalence of 16.1 patients per 100,000 in 2011 [3]. Third, in this study, we included the responses of the participants who answered more than three times in the total duration of the study to evaluate the ability to capture the changes. This implies that our study results may vary according to participants who answered less than three times or did not answer at all. A qualitative study on participants’ experience with the EMA method or research on psychometric properties of the K-PsyMate scale for different populations such as patients with other disorders and symptoms will be an interesting topic for future study.

### Conclusion

This study provides evidence that the K-PsyMate scale has good validity but poor to moderate level of agreement between the measurements. The poor to moderate level of agreement evaluated by ICCs may reflect the characteristics of the EMA data collected in real and natural living situations and environments. Our study shows that the K-PsyMate scale uploaded in a mobile app can be a valid and reliable tool for evaluating the momentary mood of patients with MMD because using a mobile app is convenient for patients as they are familiar with their own smartphones, which they use in their daily lives.

## Appendix

Multimedia Appendix 1 Characteristics of the participants and subgroup analysis of the usability of the ecological momentary assessment method by using a mobile app (n=67).

## Abbreviations

CVI: content validity index
EFA: exploratory factor analysis
EMA: ecological momentary assessment
HADS: hospital anxiety and depression scale
ICC: intraclass correlation coefficient
I-CVI: item-level content validity index
K-PsyMate: Korean version of the PsyMate
MMD: moyamoya disease
S-CVI: scale-level content validity index
