# Supplementary material for: Psychometric Properties of the Korean Version of the PsyMate Scale Using a Smartphone App: Ecological Momentary Assessment Study
Source: JMIR Mhealth Uhealth. 2020 Jul 21;8(7):e17926. doi: 10.2196/17926 (PMC7404014; doi:10.2196/17926)
Supplement: Multimedia Appendix 1 [file mhealth_v8i7e17926_app1.pdf]

**Multimedia Appendix 1. Characteristics of participants and sub-group analysis of usability of EMA method using a mobile app (n = 67)**

| Variables | n (%) | Accuracy Mean (SD) | Easiness Mean (SD) | Enjoyment Mean (SD) | Interruption Mean (SD) | Intention Mean (SD) |
|---|---|---|---|---|---|---|
| **Age groups** | | | | | | |
| 20-29 | 7 (10) | 4.50 (0.50) | 4.38 (0.56) | 3.93 (0.53) | 2.00 (1.00) | 3.50 (0.65) |
| 30-39 | 26 (39) | 4.19 (1.02) | 4.05 (0.91) | 3.31 (1.02) | 2.81 (1.06) | 3.27 (1.02) |
| 40-49 | 16 (24) | 3.94 (0.89) | 3.94 (0.95) | 3.97 (0.59) | 2.62 (0.81) | 3.34 (0.85) |
| 50-59 | 14 (21) | 4.36 (0.89) | 4.38 (0.94) | 3.93 (1.04) | 2.07 (1.00) | 3.00 (0.94) |
| ≥60 | 4 (6) | 3.13 (1.45) | 3.92 (0.74) | 3.13 (0.48) | 2.00 (1.16) | 3.25 (1.55) |
| t/F | N/A | 1.78 | 0.71 | 2.41 | 2.04 | 0.39 |
| *P* value | N/A | 0.14 | 0.59 | 0.06 | 0.10 | 0.82 |
| **Gender** | | | | | | |
| Female | 50 (75) | 4.09 (1.01) | 4.18 (0.87) | 3.69 (0.94) | 2.42 (1.03) | 3.20 (0.93) |
| Male | 17 (25) | 4.26 (0.87) | 3.94 (0.91) | 3.53 (0.86) | 2.65 (1.00) | 3.41 (1.02) |
| t/F | N/A | 0.64 | -0.96 | -0.62 | 0.79 | 0.79 |
| *P* value | N/A | 0.53 | 0.34 | 0.54 | 0.43 | 0.43 |
| **Marital status** | | | | | | |
| Married | 53 (79) | 4.08 (1.01) | 4.10 (0.94) | 3.60 (0.95) | 2.47 (1.03) | 3.23 (0.99) |
| Single | 14 (21) | 4.36 (0.82) | 4.19 (0.65) | 3.82 (0.77) | 2.50 (1.02) | 3.36 (0.82) |
| t/F | N/A | 0.96 | 0.34 | 0.79 | 0.92 | 0.46 |
| *P* value | N/A | 0.34 | 0.74 | 0.43 | 0.93 | 0.65 |
